# Discovery of region‐specific lipid biomarkers characterized by distinct changes over time in the brains of APPsw mice

**DOI:** 10.1002/alz.091901

**Published:** 2025-01-09

**Authors:** Laura Beth McIntire, Krista Minéia Wartchow, Ana Paula Costa, Jan Krumsiek, Jason Mares, Vilas Menon, Artur Lazarian, Nicholas Bartelo, William Dartora

**Affiliations:** ^1^ Weill Cornell Medical College, Brain Health Imaging Institute, Department of Radiology, New York, NY USA; ^2^ Weill Cornell Medicine, New York, NY USA; ^3^ Institute for Computational Biomedicine, Englander Institute for Precision Medicine, Department of Physiology and Biophysics, Weill Cornell Medicine, New York, NY USA; ^4^ Columbia University, New York, NY USA; ^5^ Columbia University Irving Medical Center, New York, NY USA; ^6^ Weill Cornell Medicine, Brain Health Imaging Institute, Department of Radiology, New York, NY USA; ^7^ Weill Cornell Medicine, New York City, NY USA

## Abstract

**Background:**

Alzheimer's disease (AD) is associated with impaired lipid metabolism in the brain. To identify the specific regions where pathological change to cell functionality occurs, a spatial investigation of regional lipid dysregulation is needed.

**Method:**

We measured untargeted spatial lipidomics using Desorption Electrospray Ionization (DESI) mass spectrometry in the brains of mice from two genotypes, wild type (WT) and APPsw, an AD mouse model overexpression amyloid precursor protein (APP). We generated a longitudinal profile of these mice at ages 6 months, 12 months, and 22 months. We computationally segmented brain regions defined by the Allen Mouse Brain Atlas to discover region‐specific differential lipid biomarkers across age and genotype. We developed “Spatial Lipidomics Analysis Tool” (SLAT), a computational framework which identifies statistically differential ions between age and genotype in a region‐specific manner, and applied this to our data. Independently of SLAT, we applied K‐means clustering to lipidomics as an unbiased approach to find regions in whole brains that may drive the development of disease. We applied K‐means clustering to log2 fold changes (log2FC) to find groups of significant ions reported by SLAT characterized by similar changes in abundance in APPsw mice over time compared to 6 months WT mice.

**Result:**

We identified the globus pallidus (GP) as having multiple significantly differential lipids between WT and APPsw samples. However, whole brain analysis was unable to identify significant differential ions between genotypes, demonstrating the advantage of region‐specific‐analysis. Additionally, the GP was found to drive clustering for specific ages and genotypes. Longitudinal analysis of significant GP ions revealed multiple groups of ions with coordinated changes in ion intensity over time. The group of ions with the greatest temporal changes had a 0.50 log2FC increase from 6 to 12 months, followed by a 0.63 log2FC decrease from 12 to 22 months, on average.

**Conclusion:**

Our results illustrate lipid biomarkers specific to the GP which may have potential use for the diagnosis and prognosis of AD. The development of SLAT as a partially automated pipeline to analyze longitudinal data with different phenotypes can be utilized as a tool for discovery of disease‐specific mechanisms from spatial lipidomics and metabolomics data.

